# Heart rate variability is associated with social value orientation in males but not females

**DOI:** 10.1038/s41598-018-25739-4

**Published:** 2018-05-09

**Authors:** Alexander Lischke, Anett Mau-Moeller, Robert Jacksteit, Rike Pahnke, Alfons O. Hamm, Matthias Weippert

**Affiliations:** 1 Department of Psychology, University of Greifswald, Greifswald, Germany; 2 Department of Orthopaedics, University Medicine Rostock, Rostock, Germany; 3 Department of Sport Science, University of Rostock, Rostock, Germany

## Abstract

Phylogenetic and neurobiological theories suggest that inter-individual differences in high frequency heart rate variability (HF-HRV) are associated with inter-individual differences in social behavior and social cognition. To test these theories, we investigated whether individuals with high and low HF-HRV would show different preferences for cooperative behavior in social contexts. We recorded resting state HF-HRV in 84 healthy individuals before they completed the Social Value Orientation task, a well-established measure of cooperative preferences. HF-HRV was derived from short-term (300 s) and ultra-short-term (60 s, 120 s) recordings of participants’ heart rate to determine the robustness of possible findings. Irrespective of recording length, we found a sex-dependent association between inter-individual differences in HF-HRV and inter-individual differences in social value orientation: The preference for cooperation was more pronounced among individuals with high as compared low HF-HRV, albeit only in male and not in female participants. These findings suggest that males with high HF-HRV are more inclined to engage in cooperative behavior than males with low HF-HRV.

## Introduction

Across industrialized and non-industrialized societies, humans show a remarkable and distinct suite of cooperative behavior^1–4^. Humans often cooperate with one another, even if cooperation is costly for the self and beneficial for the other^5,6^. Cooperation usually emerges during interactions with well-known individuals because cooperative behavior is more expected, reinforced and rewarded among well-known than among less known individuals^7–11^. However, cooperation also occurs among unknown individuals, which do not necessarily expect, reinforce or reward cooperative behavior^7–11^. Individuals may, thus, have a preference for cooperation, independent of considerations regarding the expectancy, reinforcement or reward of cooperation, which is driving their behavior during social interactions^12^. As an individual’s preference for cooperation appears to affect cooperative behavior in various contexts^13–19^, there is a growing interest for biomarkers indicating whether an individual is more likely to cooperate or defect during social interactions.

Vagally mediated heart rate variability (vmHRV), a measure of parasympathetic induced changes in heart rate [HR]^20^, has been suggested to be a promising biomarker for an individual’s behavior in social contexts^21,22^. An individual’s social behavior is orchestrated by a network of prefrontal and para-limbic brain regions that are relevant for a plethora of social processes^23^, implying that functional and structural changes in these brain regions are associated with changes in social cognition and social interaction. As functional and structural changes in these brain regions are also associated with changes in vmHRV^22,24,25^, changes in vmHRV reflect changes in an individual’s social behavior. Inter-individual differences in vmHRV have, therefore, been suggested to indicate inter-individual differences in social cognition and social interaction^21,22^. Accordingly, there are marked differences in social cognition and social interaction between individuals that show differences in vmHRV^21,22^. For instance, individuals with high vmHRV are more likely to recognize facial expressions of others^26–28^, to regulate emotional responses towards others^29–31^ and to establish cooperative relationships with others^32–34^ than individuals with low vmHRV. The difference in cooperative behavior between individuals with high and low vmHRV may be due to different preferences for cooperation^32,33,35^, suggesting a more pronounced preference for and display of cooperation among individuals with high than low vmHRV.

**Figure 1:**
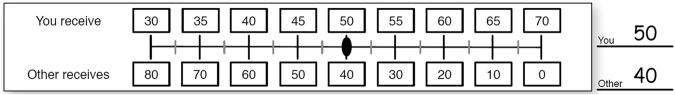
Example of a continuum of self/other pay off allocations that are used in the Social Value Orientation Task [SVO]^36^.

To investigate whether inter-individual differences in vmHRV would be associated with inter-individual differences in cooperation, we assessed vmHRV and cooperative preferences in a relatively large sample of participants. Inter-individual differences in vmHRV were determined on basis of short-term (300 s) and ultra-short-term (120 s, 60 s) resting state recordings of participants’ HR. Whereas the use of short-term measures of vmHRV is recommended and well established^20^, the use of ultra-short-term measures of vmHRV is less established because it is currently unknown whether these measures are similarly associated with inter-individual differences in social cognition and social interaction as short-term measures. Using short-term and ultra-short-term measures of vmHRV allowed us to investigate whether the association between inter-individual differences in vmHRV and inter-individual differences in cooperative preferences would be invariant across recording conditions. The use of these measures, thus, helped us to assess the robustness of the association between inter-individual differences in vmHRV and inter-individual differences in cooperative preferences. Inter-individual differences in cooperative preferences were determined with the Social Value Orientation task [SVO]^36^, an established resource allocation task that differentiates between cooperative and non-cooperative allocations on basis of participants’ choices over a continuum of self/other payoff allocations (see Fig. 1). The SVO differs markedly from other resource allocation tasks, including those that have been used in comparable studies^32,33,35^, because participants’ cooperative or non-cooperative allocations are not affected by strategic considerations^36^. Participants’ allocations on the SVO, thus, represent an unbiased measure of their preference for cooperation. Using the SVO^36^, we expected to find the preference for cooperative allocations to be more pronounced among participants with high than low vmHRV. We also expected that inter-individual differences in cooperative preferences would be mediated by participants’ sex because male and female participants perform differently on resource allocation tasks that measure inter-individual differences in cooperation^37,38^.

## Method

### Participants

Prior to participant recruitment, we performed a power analysis to determine the number of participants that we needed to detect a meaningful association between inter-individual differences in vmHRV and inter-individual differences in cooperative preferences. Due to a lack of previous studies investigating the association of inter-individual differences in vmHRV with inter-individual differences in cooperative *preferences*, we turned to studies that investigated the association of inter-individual differences in vmHRV with inter-individual differences in cooperative *behavior*^32,33^. As these studies revealed medium-to-large sized associations (*r* = 0.35, *f* = 0.37)^32,33^, we expected to find an association of similar size in our study. G*Power^39^ indicated that we had to recruit 82 participants to be able to detect such an association in dimensional and categorical analyses [*α* = 0.05, 1-*β* = 80, *r* = 0.35, *f* = 0.35]. Slightly over-recruiting, we included 84 participants, 41 males and 43 females, in our study. In order to be included in the study, participants had to be aged between 18 and 35 years and to be native speakers. Participants with mental disorders and participants who were in psychotherapeutic treatment were excluded from the study. Of the 84 participants, 8 participants did not provide valid data due to equipment dysfunction (*n* = 1) or misunderstanding of the task instructions (*n* = 7). Consequently, the data of 76 participants, 39 males and 37 females, were considered in the analyses (see Table 1).

**Table 1:** Participant characteristics.

	Male participants				Female participants				Test statistic		
	*M*	*SD*	*Min*	*Max*	*M*	*SD*	*Min*	*Max*	*F*(1,74)/*F*(1,59)	*p*	*η*_*p*_^2^
Age	26.49	4.58	18	35	25.81	3.61	20	34	0.508	0.478	0.007
Heart rate variability											
Log-HF-HRV-300	2.71	0.54	1.23	3.75	2.79	0.41	1.71	3.65	0.528	0.470	0.007
Log-HF-HRV-120	2.76	0.59	1.17	4.12	2.82	0.46	1.93	3.82	0.260	0.611	0.004
Log-HF-HRV-60	2.85	0.60	1.20	4.03	2.86	0.42	1.95	4.12	0.007	0.936	0.000
Social value orientation											
SVO angle^a^	32.83	11.05	0.00	61.39	32.00	10.89	−3.81	50.83	0.108	0.743	0.000
IA index^b^	0.31	0.32	0.05	1.00	0.11	0.11	0.00	0.43	10.370	0.002**	0.149

*Note*. Log-HF-HRV-300 = log-transformed high frequency heart rate variability derived from short-term HR recordings (300 s), Log-HF-HRV-120 = log-transformed high frequency heart rate variability derived from ultra-short-term HR recordings (120 s), Log-HF-HRV-60 = log-transformed high frequency heart rate variability derived from ultra-short-term HR recordings (60 s), SVO angle = Social Value Orientation angle^36^, IA Index = Inequality Aversion Index^36^.

^a^Data on SVOA angle was available for 76 participants, 37 females and 39 males.

^b^Data on IA index was only available for 61 participants, 30 females and 31 males because these were the only participants that met the conditions for the determination of the IA index^36^.

**p ≤ 0.01.

All participants provided written-informed consent before taking part in the study and were fully debriefed after completion of the study. The study was approved by the ethics committee of the University of Rostock and carried out in accordance with the Declaration of Helsinki.

### Procedure

After arrival at the laboratory, participants were asked to use the bathroom to control for the effects of bladder filling and gastric distension on vmHRV^40^. Participants were then seated in a comfortable chair and prepared for a resting state recording of their HR. In line with recent recommendations^41^, participants were instructed to sit still, to breathe spontaneously and to keep their eyes open during the HR recording. Immediately after receiving the instructions, participants’ HR was recorded for a total duration of 5 min (300 s). Thereafter, participants completed the SVO task^36^.

### Heart rate variability

HR was continuously recorded with the RS800 (Polar Electro Oy, Kempele, Finland), a mobile HR monitor that has been shown to record participants’ HR as accurate as conventional HR monitors^42,43^. The RS800 comprises a two-lead chest belt system for data recording (sample rate: 1000 Hz) and a wrist watch for data storage. Device specific software (Polar ProTrainer 5; Polar Electro Oy, Kempele, Finland) was used to transfer the recorded data from the wrist watch to a computer for data processing with Kubios HRV 2.2^44^. Following established guidelines^20^, the recorded data was detrended (smoothn priors: *λ* = 500), visually inspected and, whenever necessary, artifact corrected with adaptive filtering (cubic spline interpolation). Overall, less than 5% of participants’ data had to be corrected for artifacts, indicating the reliability and validity of the HR recording. After artifact correction, the recorded data was subjected to a spectral analysis (Fast Fourier Transformation; Welch’s periodogram: 256 s window with 50% overlap) to determine HF-HRV (0.15–0.4 Hz) as outlined in established guidelines^20^. HF-HRV was determined for different recording lengths^45^, including a short-term recording (300 s from the start of the recording session, HF-HRV-300) and two ultra-short-term recordings (60 s from the start of the recording session, HF-HRV-60; 120 s from the start of the recording session, HF-HRV-120). In contrast to other HRV measures, HF-HRV is a robust measure of changes in HR that are mediated by the vagus nerve^20^.

### Social Value Orientation

The SVO task, an established resource allocation task^36^, comprised 6 primary and 9 secondary items with a choice over a defined continuum of self/other payoff allocations (see Fig. 1). Participants had to choose the allocation that reflected their most preferred joint payoffs for themselves and another participant whose identity remained anonymous throughout the study. Participants’ choices on the primary items were used to determine an index of participants’ social value orientation, the SVO angle^36^. Higher angular degrees on the SVO angle indicated participants’ preference for cooperative allocations (i.e., pro-social allocations, allocations with higher payoffs for the other than for the self) as compared to non-cooperative allocations (i.e., pro-self allocations, allocations with lower payoffs for the other than for the self). To further describe participants’ preference for cooperative allocations, participants’ choices on the secondary items were used to compute another index of participants’ social value orientation, the inequality-aversion index [IA index^36^. An index of 1 indicated that participants’ preference for cooperative allocations was driven by the motivation of joint maximization (i.e., maximizing payoffs for both, the self and the other), whereas an index of 0 indicated that participants’ preference for cooperative allocations was driven by the motivation of inequality aversion (i.e., minimizing differences between payoffs for the self and payoffs for the other).

### Statistical Analysis

SPSS 22 (SPSS Inc., Chicago, IL, USA) was used for all analyses. Preliminary analyses were performed to investigate the characteristics of male and female participants. To this end, analyses of variances (ANOVAs) were used to compute differences in age (years), social value orientation (SVO angel, IA index) and HF-HRV (HF-HRV-300, HF-HRV-120, HF-HRV-60) between male and female participants. Main analyses were performed to investigate the association between inter-individual differences in HF-HRV and inter-individual differences in social value orientation among male and female participants. These analyses comprised categorical and dimensional analyses. For the categorical analyses, two-way ANOVAs (Sex × Group) were used to compute differences in social value orientation (SVO angle, IA index) between male and female participants with high and low HF-HRV on basis of a median-split. For the dimensional analyses, correlations between HF-HRV and social value orientation (SVO angle, IA index) were computed, separately for male and female participants. In addition, Fisher’s z-transformation^46^ was used to compare the respective correlation coefficients with one another. To assess the robustness of the categorical and dimensional analyses, the analyses were performed for HF-HRV measures that were derived from short-term (HF-HRV-300) and ultra-short-term (HF-HRV-120, HF-HRV-60) recordings of participants’ HR. Correspondence between short-term and ultra-short-term measures of HF-HRV was determined on basis of intra-class correlations (ICC: absolute agreement, two-way ANOVA^47^). Prior to all analyses, HF-HRV was log transformed (log 10) to account for deviations from normality distribution. The significance level for all analyses was set at *p* ≤ 0.05. In addition to the significance level (*p*), effect size measures (*η*_*p*_^2^*, r* and *q*) were determined to facilitate the interpretation of (marginally) significant findings^48^.

## Results

### Participant characteristics

A one-way ANOVA revealed no age differences between male and female participants [*F*(1,74) = 0.508, *p* = 0.478, *η*_*p*_^2^ = 0.007; see Table 1]. A series of further one-way ANOVAs also revealed no differences in HF-HRV between male and female participants, regardless whether HF-HRV was derived from short-term or ultra-short-term recordings of participants’ HR [*F* ≤ 0.528, *p* ≥ 0.470, *η*_*p*_^2^ ≤ 0.004; see Table 1]. Another series of one-way ANOVAs indicated differences in IA index [*F*(1,59) = 10.370, *p* = 0.002, *η*_*p*_^2^ = 0.149; see Table 1] but not in SVO angle [*F*(1,74) = 0.180, *p* = 0.743, *η*_*p*_^2^ = 0.000; see Table 1] between male and female participants. Female participants showed a lower IA index than male participants.

### Short-term measures of heart rate variability and measures of social value orientation

**Figure 2:**
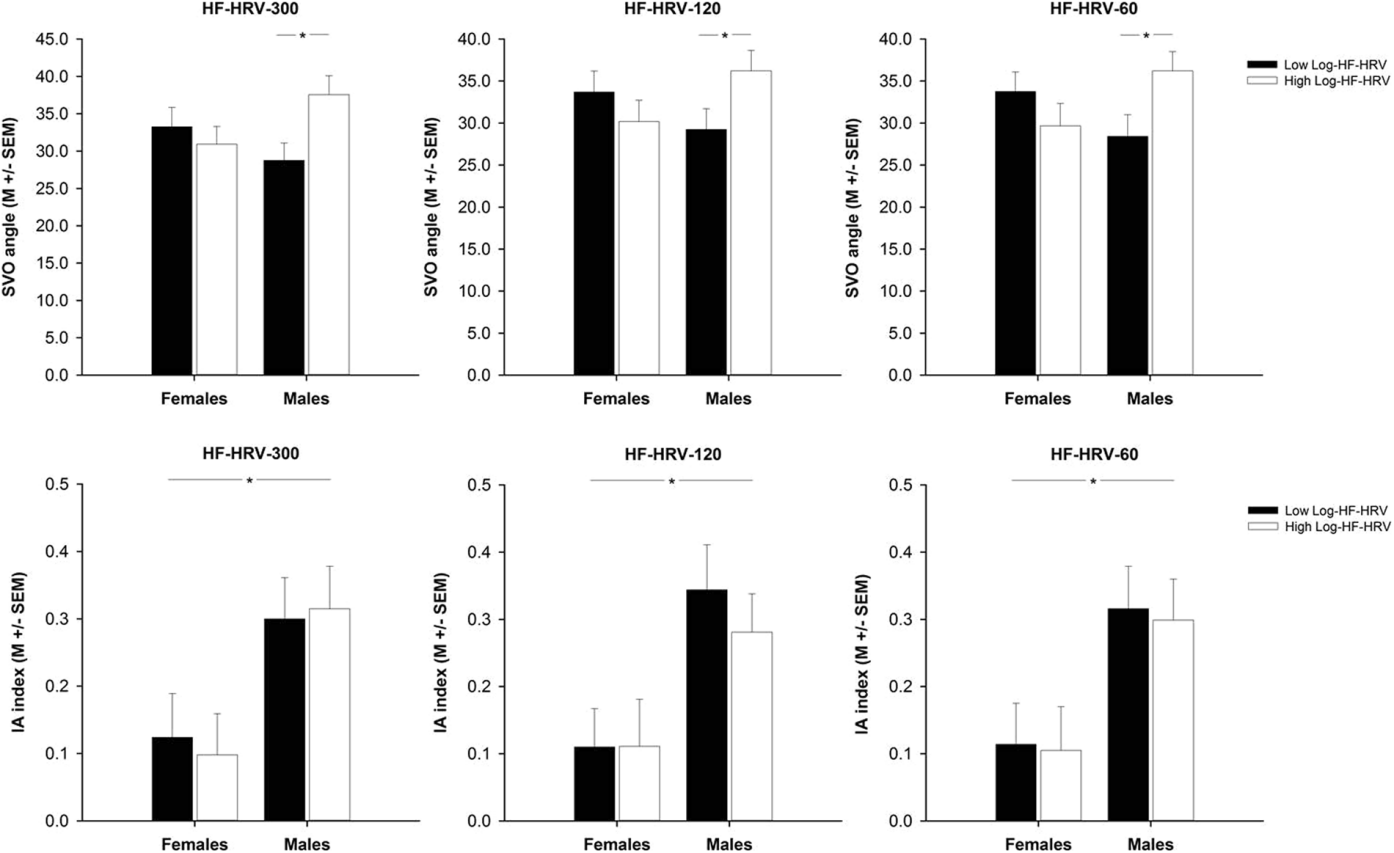
Barplots demonstrating differences in social value orientation (SVO angle, IA index) between participants with high (white bars) and low (black bars) log-transformed high-frequency heart rate variability (Log-HF-HRV) that was derived from short-term (300 s) or ultra-short-term (120 s, 60 s) recordings of male and female participants’ heart rate. Bars represent mean values (M) and error bars represent standard error of mean values (SE M). *p ≤ 0.05.

**Figure 3:**
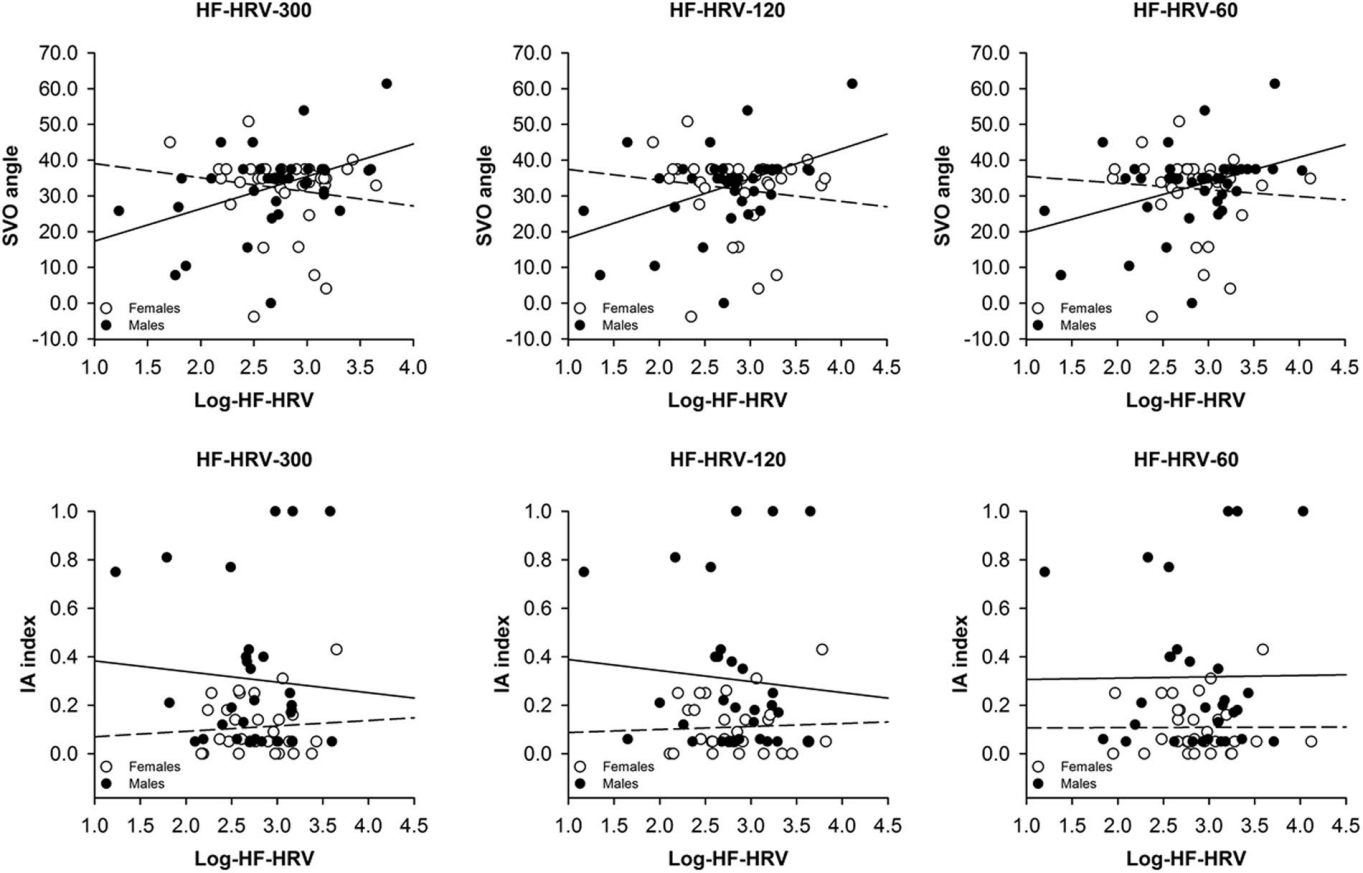
Scatterplots with lines of best fit demonstrating correlations between social value orientation (SVO angle, IA index) and log-transformed high frequency heart rate variability (Log-HF-HRV) that was derived from short-term (300 s) or ultra-short-term (120 s, 60 s) recordings of male (black circles, solid lines) and female (white circles, dotted lines) participants’ heart rate.

A two-way ANOVA (Sex × Group) indicated sex-specific differences in SVO angle between male and female participants with low and high HF-HRV [all effects of sex or group: *F* ≤ 1.752, *p* ≥ 0.190, *η*_*p*_^2^ ≤ 0.024; interaction of sex and group: *F*(1,72) = 5.219, *p* = 0.025, *η*_*p*_^2^ = 0.068; see Table S1 and Fig. 2]. Follow-up one-way ANOVAs revealed differences in SVO angle between male participants with high and low HF-HRV [*F*(1,37) = 7.167, *p* = 0.011, *η*_*p*_^2^ = 0.162; see Fig. 2] but not between female participants with high and low HF-HRV [*F*(1,35) = 0.420, *p* = 0.521, *η*_*p*_^2^ = 0.012; see Fig. 2]. SVO angle was higher in male participants with high HF-HRV than in male participants with low HF-HRV. Accordingly, there was a correlation between HF-HRV and SVO angle in male [*r*(39) = 0.349, *p* = 0.030; see Table S2 and Fig. 3] but not in female [*r*(37) = −0.195, *p* = 0.247; see Table S2 and Fig. 3] participants as indicated by a series of correlation analyses. A direct comparison of the respective correlation coefficients confirmed that HF-HRV was positively correlated with SVO angle in male but not in female participants [*z* = 2.349, *p* = 0.019, *q* = 0.562; see Fig. 3].

A two-way ANOVA (Sex × Group) indicated sex-specific differences in IA index between male and female participants that did not depend on participants’ HF-HRV [effect of sex: *F*(1,57) = 9.948, *p* = 0.003, *η*_*p*_^2^ = 0.149; all other effects and interactions of sex or group: *F* ≤ 0.105, *p* ≥ 0.747, *η*_*p*_^2^ ≤ 0.002; see Table S1 and Fig. 2]. Female participants showed a lower IA index than male participants. A series of correlation analyses revealed no correlations between HF-HRV and IA Index in male or female participants [*r* ≤ −0.090, *p* ≥ 0.634; see Table S2 and Fig. 3].

### Ultra-short-term measures of heart rate variability and measures of social value orientation

A series of two-way ANOVAs (Sex × Group) confirmed the aforementioned sex-specific differences in SVO angle between participants with low and high HF-HRV [all effects involving sex or group: *F*(1,72) ≤ 0.573, *p* ≥ 0.452, *η*_*p*_^2^ ≤ 0.008; all interactions of sex and group: *F*(1,72) ≥ 4.527, *p* ≤ 0.037, *η*_*p*_^2^ ≥ 0.059; see Table S1 and Fig. 2]. Follow-up one-way ANOVAs revealed, again, a higher SVO angle in male participants with high HF-HRV than in male participants with low HF-HRV [*F*(1,37) ≥ 4.219, *p* ≤ 0.047, *η*_*p*_^2^ ≥ 0.102; see Fig. 1] and a similar SVO angle in female participants with high and low HF-HRV [*F*(1,35) ≤ 1.270, *p* ≥ 0.267, *η*_*p*_^2^ ≤ 0.035; see Fig. 1]. Correlation analyses confirmed the aforementioned pattern of correlations between HF-HRV and SVO angle: There was, again, a positive correlation between HF-HRV and SVO angle in male [*r*(39) ≥ 0.333, *p* ≤ 0.038; see Table S2 and Fig. 3] but not in female [*r*(37) ≤ −0.256, *p* ≥ 0.126; see Table S2 and Fig. 3] participants as indicated by a direct comparison of the respective correlation coefficients [*z* ≥ 2.309, *p* ≤ 0.021, *q* ≥ 0.552; see Table S2 and Fig. 3].

A two-way ANOVA (Sex × Group) confirmed the aforementioned sex-specific differences in IA index between male and female participants that were independent of participants’ HF-HRV [all effects of sex: *F*(1,57) ≥ 10.060, *p* ≤ 0.002, *η*_*p*_^2^ ≥ 0.150; all other effects and interactions of sex or group: *F*(1,57) ≤ 0.260, *p* ≥ 0.612, *η*_*p*_^2^ ≤ 0.005; see Table S1 and Fig. 2]. Female participants showed, again, a lower IA index than male participants. A series of correlation analyses confirmed that there were no correlations between HF-HRV and IA Index in male or female participants [all *r* ≤ −0.119, all *p* ≥ 0.524; see Table S2 and Fig. 3].

### Short-term and ultra-short-term measures of heart rate variability

A series of intra-class correlation analyses was performed to investigate the correspondence between HF-HRV measures that were derived from short-term and ultra-short term recordings of participants’ HR. According to these analyses, there was a high correspondence between the different HF-HRV measures among male as well as female participants [all ICC ≥ 0.878; see Table 2).

**Table 2:** Intra-class correlations between short-term and ultra-short-term measures of heart rate variability.

	Female participants		Male participants	
	ICC	95% CI	ICC	95% CI
Log-HF-HRV-300 vs.Log-HF-HRV-120	0.946	[0.896, 0.972]	0.961	[0.926, 0.980]
Log-HF-HRV-300 vs. Log-HF-HRV-60	0.878	[0.764, 0.937]	0.931	[0.829, 0.968]
Log-HF-HRV-120 vs. Log-HF-HRV-60	0.974	[0.935, 0.988]	0.949	[0.901, 0.974]

*Note*. ICC = intra-class correlations, 95% CI = 95% confidence interval, Log-HF-HRV-300 = log-transformed high frequency heart rate variability derived from short-term HR recordings (300 s), Log-HF-HRV-120 = log-transformed high frequency heart rate variability derived from ultra-short-term HR recordings (120 s), Log-HF-HRV-60 = log-transformed high frequency heart rate variability derived from ultra-short-term HR recordings (60 s).

## Discussion

In the present study, we investigated whether inter-individual differences in vmHRV would be associated with inter-individual differences regarding the preference for cooperation. We found a sex-dependent association between inter-individual differences in vmHRV and inter-individual differences in cooperative preferences. Male participants with high vmHRV showed more cooperative preferences than male participants with low vmHRV. Female participants with low and high vmHRV, on the contrary, did not differ in their cooperative preferences. Considering that inter-individual differences in cooperative preferences as well as inter-individual differences in vmHRV were more pronounced among male than female participants (see Table 1 and Fig. 3 for a range of the respective values that was larger in male than female participants), it was more likely that an association between inter-individual differences in cooperative preferences and inter-individual differences in vmHRV emerged in analyses involving male rather than female participants. However, this association only emerged in analyses of participants’ SVO angle but not in analyses of participants’ IA index. Analyses of participants’ IA index are generally more complicated than analyses of participants’ SVO angle because the determination of the IA index depends on more conditions than the determination of the SVO angle^36^. As some participants did not meet the conditions for these types of analyses, fewer participants were considered in the analyses of the IA index than in the analyses of the SVOA angle. Differences in statistical power may, thus, have accounted for the inconsistency of findings. The findings of the analyses regarding participants’ IA index should, therefore, be treated with caution, until replicated and extended in future studies. For this reason, we centered our discussion on the findings that emerged in the analyses of participants’ SVO angle rather than on the findings that emerged in the analyses of participants’ IA index. In this respect, it is important to note that it did not matter whether short-term or ultra-short-term measures of vmHRV were considered in these analyses. Moreover, short-term and ultra-short-term measure of vmHRV were substantially associated with one another, replicating and extending findings of a previous study whose focus was not on frequency domain measures of vmHRV as in the present study but on time domain measures of vmHRV^45^. Taken together, these findings suggest an a robust and substantial association between inter-individual differences in vmHRV and inter-individual differences regarding cooperative preferences in male as compared to female participants.

Inter-individual differences in vmHRV are associated with various aspects of social behavior, which may help to understand why inter-individual differences in vmHRV account for inter-individual differences in cooperative preferences and cooperative behavior. As individuals with high vmHRV are more successful in regulating emotional responses towards others than individuals with low vmHRV^29–31^, they may be less stressed and more relaxed during social interactions^49–51^. Due to the absence of feelings of stress, individuals with high vmHRV may be more motivated to engage in cooperative behavior than individuals with low vmHRV^32,33^, which may be reflected in the respective differences regarding individuals’ preference for cooperation. Moreover, by displaying cooperative behavior^32,33^, individuals with high vmHRV may have less difficulties to initiate and maintain social relationship than individuals with low vmHRV^49,50^. This may explain why individuals with high vmHRV report more feelings of connectedness and comfort in social contexts than individuals with low vmHRV^34,49–51^. Of note, individuals suffering from mental disorders that are characterized by discomfort in social contexts and impairments in social interactions often display lower vmHRV than healthy individuals^52,53^. For instance, depressed individuals, which are known to display lower vmHRV than non-depressed individuals^54,55^, have marked difficulties in sustaining cooperative relationships^56^. Inter-individual differences in vmHRV may, thus, account for inter-individual differences regarding the preference for and display of cooperative behavior in healthy as well as mentally-disordered individuals, indicating that vmHRV may indeed be a biomarker for social behavior^21,22^. Future studies investigating the association between inter-individual differences in vmHRV and inter-individual differences in cooperation in healthy as well as in mentally-disordered individuals may help to determine whether vmHRV qualifies as biomarker for social behavior.

The neurobiological mechanisms mediating the association between inter-individual differences in vmHRV and inter-individual differences in cooperation may be best elucidated by comparing the neurobiological mechanisms underlying changes in vmHRV with the neurobiological mechanisms underlying changes in cooperation. Changes in cooperative preferences and cooperative behavior are mediated by a network of prefrontal, para-limbic and meso-limbic brain regions^57,58^. Of these brain regions, prefrontal and para-limbic ones are of particular relevance because functional and structural changes in these brain regions lead to profound changes regarding the preference for^59–62^ and the display of ^63–69^ cooperative behavior. Prefrontal and para-limbic brain regions are also part of a network of brain regions that mediate changes in vmHRV^22,24,25^. As functional^70^ and structural^71^ changes in these brain regions are closely associated with changes in vmHRV, changes in vmHRV may reflect changes in cooperative preferences and cooperative behavior that are due to functional and structural changes in the aforementioned network of brain regions. It may, thus, be conceivable that the preference for and the display of cooperative behavior is more pronounced among individuals with high than low vmHRV because individuals with high HRV are more efficient in recruiting the relevant brain regions driving cooperation in social contexts. In this regard, it is noteworthy that individuals suffering from mental disorders that are associated with deficits in social behavior show alterations in vmHRV^52,53^ as well as alterations in networks of brain regions that are centered on prefrontal and para-limbic brain regions^72^. Inter-individual differences in vmHRV may, thus, indicate inter-individual differences regarding the engagement of brain regions that mediate cooperative preferences and cooperative behavior in healthy as well as mentally-disordered individuals, implying once more that vmHRV may function as a biomarker for social behavior^21,22^. Future studies should consider behavioral and neural measures in their analyses on the association between inter-individual differences in vmHRV and inter-individual differences in cooperation to determine whether vmHRV has the potential to work as a biomarker for social behavior.

The present study does not only help to elucidate the neurobiological mechanisms underlying cooperation in social contexts, but also suggest some methodological modifications that may be helpful for further studies investigating the association of inter-individual differences in vmHRV with inter-individual differences in cooperative preferences and cooperative behavior. First of all, inter-individual differences in cooperation do not necessarily have to be assessed with complex tasks, like, for example, a re-iterated trust game^10^. Simpler tasks, such as the SVO, which has excellent psychometric properties^36^, may be sufficient to differentiate between cooperative and non-cooperative individuals. The SVO does not require real or simulated interactions between participants, indicating that the administration of the SVO is less time- and resource-consuming than the administration of more complex tasks^36^. Second, it may be sufficient to assess inter-individual difference in vmHRV on basis of ultra-short-term instead of short-term HR recordings. There was not only a remarkable correspondence between ultra-short-term and short-term masures of vmHRV regarding the measurement of inter-individual differences in vmHRV but also regarding the measurement of the association between inter-individual differences in vmHRV and inter-individual differences in cooperative preferences, replicating and extending the findings of a previous study^45^. Ultra-short-term measures of vmHRV, thus, represent reliable and valid alternatives to short-term measures of vmHRV, indicating a need to revise current guidelines that recommend the use of short-term measures over the use of ultra-short term measures^20^. Using ultra-short-term instead of short-term measures may save time and resources during the assessment of vmHRV, which has been considered as a simple measure regarding the engagement of prefrontal and para-limbic brain regions during the regulation of social processes^21,22^. Combining ultra-short-term measures of vmHRV, such as HF-HRV-120, with short measures of cooperation, such as the SVO, may, thus, be interesting for researchers that need to investigate the neurobiological basis of inter-individual differences in cooperation in a time- and resource-efficient manner. In particular, researchers investigating biomarkers for social behavior in large-scale studies, such as genome-wide association studies, may benefit from the combined use of the aforementioned measures when time and resources are scarce.

Overall, the findings of the present study indicate that inter-individual differences in vmHRV are associated with inter-individual differences in cooperative preferences, albeit only in males and not in females. These findings are consistent with those of previous studies that revealed an association between inter-individual differences in vmHRV and inter-individual differences in cooperative behavior. Taken together, these findings support phylogenetic and neurobiological theories that suggest that vmHRV may work as a biomarker for various social processes^21,22^, including those that are related to cooperative preferences and cooperative behavior.

## Electronic supplementary material

Supplementary Material
