# Supplementary material for: Heart rate variability is associated with social value orientation in males but not females
Source: Sci Rep. 2018 May 9;8:7336. doi: 10.1038/s41598-018-25739-4 (PMC5943302; doi:10.1038/s41598-018-25739-4)
Supplement: Supplementary file 1 — Supplementary Material [file 41598_2018_25739_MOESM1_ESM.docx]

**Heart rate variability is associated with social value orientation in males but not females**

Alexander Lischke^1^, PhD; Anett Mau-Moeller^2^, PhD; Robert Jacksteit^2^, PhD; Rike Pahnke^3^; Alfons O. Hamm^1^, PhD; Matthias Weippert^3^, PhD

^1^Department of Psychology, University of Greifswald, Greifswald, Germany

^2^Department of Orthopaedics, University Medicine Rostock, Rostock, Germany

^3^Department of Sport Science, University of Rostock, Rostock, Germany

**Supplemental Results**

**Table S1**

*Sex-dependent differences in short-term or ultra-short term measures of heart rate variability and measures of social value orientation*

|  | | Male participants | | | | |  | Female participants | | | | |  | Test statistic | | | | | | | | | | |
| --- | --- | --- | --- | --- | --- | --- | --- | --- | --- | --- | --- | --- | --- | --- | --- | --- | --- | --- | --- | --- | --- | --- | --- | --- |
|  | | High  HF-HRV | |  | Low  HF-HRV | |  | High  HF-HRV | |  | Low  HF-HRV | |  | Effect of sex | | |  | Effect of group | | |  | Interaction of  sex and group | | |
|  | | *M* | *SD* |  | *M* | *SD* |  | *M* | *SD* |  | *M* | *SD* |  | *F(1,72) / F(1,54)* | *p* | *η^2^* |  | *F(1,72) / F(1,54)* | *p* | *η_p_^2^* |  | *F(1,72) / F(1,54)* | *p* | *η_p_^2^* |
| Log-HF-HRV-300 | |  |  |  |  |  |  |  |  |  |  |  |  |  |  |  |  |  |  |  |  |  |  |  |
|  | SVO angle^a^ | 37.57 | 8.08 |  | 28.76 | 11.79 |  | 30.92 | 10.09 |  | 33.27 | 11.96 |  | 0.193 | .662 | .003 |  | 1.752 | .190 | .024 |  | 5.219 | .025* | .068 |
|  | IA index^b^ | 0.31 | 0.37 |  | 0.30 | 0.27 |  | 0.10 | 0.12 |  | 0.12 | 0.10 |  | 9.948. | 003** | .149 |  | 0.008 | .929 | .000 |  | 0.105 | .747 | .002 |
| Log-HF-HRV-120 | |  |  |  |  |  |  |  |  |  |  |  |  |  |  |  |  |  |  |  |  |  |  |  |
|  | SVO angle^a^ | 36.23 | 8.43 |  | 29.25 | 12.51 |  | 30.19 | 10.40 |  | 33.71 | 11.35 |  | 0.102 | .751 | .001 |  | 0.492 | .485 | .007 |  | 4.527 | .037* | .059 |
|  | IA index^b^ | 0.30 | 0.36 |  | 0.32 | 0.28 |  | 0.11 | 0.13 |  | 0.11 | 0.10 |  | 10.060 | .002** | .150 |  | 0.046 | .831 | .001 |  | 0.004 | .949 | .000 |
| Log-HF-HRV-60 | |  |  |  |  |  |  |  |  |  |  |  |  |  |  |  |  |  |  |  |  |  |  |  |
|  | SVO angle^a^ | 36.22 | 8.03 |  | 28.43 | 13.00 |  | 29.69 | 10.89 |  | 33.75 | 10.82 |  | 0.060 | .807 | .001 |  | 0.573 | .452 | .008 |  | 5.767 | .019* | .074 |
|  | IA index^b^ | 0.28 | 0.34 |  | 0.34 | 0.29 |  | 0.11 | 0.13 |  | 0.11 | 0.10 |  | 10.276 | .002** | .153 |  | 0.243 | .624 | .004 |  | 0.260 | .612 | .005 |
| *Note.* Log-HF-HRV-300 = log-transformed high frequency heart rate variability derived from short-term HR recordings (300 s), Log-HF-HRV-120 = log-transformed high frequency heart rate variability derived from ultra-short-term HR recordings (120 s), Log-HF-HRV-60 = log-transformed high frequency heart rate variability derived from ultra-short-term HR recordings (60 s), SVO angle = Social Value Orientation angle [[1](#_ENREF_1)], IA Index = Inequality Aversion Index [[1](#_ENREF_1)]. ^a^ Data on SVOA angle was available for 76 participants, 37 females and 39 males. ^b^ Data on IA index was only available for 61 participants, 30 females and 31 males because these were the only participants that met the conditions for the determination of the IA index [[1](#_ENREF_1)]. * *p* ≤ .05, *** p* ≤ .01. | | | | | | | | | | | | | | | | | | | | | | | | |

**Table S2**

*Sex-dependent correlations between short-term or ultra-short term measures of heart rate variability and measures of social value orientation*

|  | | Male participants | |  | | | | | Female participants | | | |
| --- | --- | --- | --- | --- | --- | --- | --- | --- | --- | --- | --- | --- |
|  | | *r*(39) */ r*(31) | *p* | | |  | | *r*(37) */ r*(30) | | | *p* | |
| Log-HF-HRV-300 | |  |  | | |  | |  | | |  | |
|  | SVO angle^a^ | .349 | .030* | |  | | -.195 | | | .247 | |  |
|  | IA index^b^ | -.084 | .651 | |  | | -.090 | | | .634 | |  |
| Log-HF-HRV-120 | |  |  | |  | |  | | |  | |  |
|  | SVO angle^a^ | .333 | .038* | |  | | -.256 | | | .126 | |  |
|  | IA index^b^ | -.119 | .524 | |  | | -.096 | | | .614 | |  |
| Log-HF-HRV-60 | |  |  | |  | |  | | |  | |  |
|  | SVO angle^a^ | .355 | .026* | |  | | -.179 | | | .289 | |  |
|  | IA index^b^ | -.069 | .711 | |  | | -.079 | | | .678 | |  |
| *Note.* Log-HF-HRV-300 = log-transformed high frequency heart rate variability derived from short-term HR recordings (300 s), Log-HF-HRV-120 = log-transformed high frequency heart rate variability derived from ultra-short-term HR recordings (120 s), Log-HF-HRV-60 = log-transformed high frequency heart rate variability derived from ultra-short-term HR recordings (60 s), SVO angle = Social Value Orientation angle [[1](#_ENREF_1)], IA Index = Inequality Aversion Index [[1](#_ENREF_1)]. ^a^ Data on SVOA angle was available for 76 participants, 37 females and 39 males. ^b^ Data on IA index was only available for 61 participants, 30 females and 31 males because these were the only participants that met the conditions for the determination of the IA index [[1](#_ENREF_1)]. ** p* ≤ .05 | | | | | | | | | | | |  |

**Supplemental References**

1. Murphy, R.O., Ackermann, K.A. & Handgraaf, M.J.J., Measuring social value orientation*.* *Judgm Decis Mak* **6** 771–81 (2011).
